# Attentional variability as a cognitive marker of clinical activity in Crohn’s disease

**DOI:** 10.1093/crocol/otag099

**Published:** 2026-09-08

**Authors:** Andreza Maia, Juliana Janeiro Schmidt, Carlos Eduardo Brandão Mello, Guilherme Janeiro Schmidt, Yolanda Faia Manhães Tolentino, Márcia Maria Amendola Pires, Ana Caroliny da Silva, Julia Dias Guimarães Silva, Marcia Lyrio Sindorf, Sergio Luis Schmidt

**Affiliations:** Pos Graduate Program in Neurology (PPGNEURO), Federal University of the State of Rio de Janeiro (UNIRIO), Rio de Janeiro, Brazil; Pos Graduate Program in Neurology (PPGNEURO), Federal University of the State of Rio de Janeiro (UNIRIO), Rio de Janeiro, Brazil; Pos Graduate Program in Neurology (PPGNEURO), Federal University of the State of Rio de Janeiro (UNIRIO), Rio de Janeiro, Brazil; Department of Gastroenterology, Gaffrée and Guinle University Hospital (HUGG), Federal University of the State of Rio de Janeiro (UNIRIO), Rio de Janeiro, Brazil; Pos Graduate Program in Neurology (PPGNEURO), Federal University of the State of Rio de Janeiro (UNIRIO), Rio de Janeiro, Brazil; Department of Gastroenterology, Gaffrée and Guinle University Hospital (HUGG), Federal University of the State of Rio de Janeiro (UNIRIO), Rio de Janeiro, Brazil; Department of Gastroenterology, Gaffrée and Guinle University Hospital (HUGG), Federal University of the State of Rio de Janeiro (UNIRIO), Rio de Janeiro, Brazil; Undergraduate Research Program, Gaffrée and Guinle University Hospital (HUGG), Federal University of the State of Rio de Janeiro (UNIRIO), Rio de Janeiro, Brazil; Undergraduate Research Program, Gaffrée and Guinle University Hospital (HUGG), Federal University of the State of Rio de Janeiro (UNIRIO), Rio de Janeiro, Brazil; Department of Gastroenterology, Gaffrée and Guinle University Hospital (HUGG), Federal University of the State of Rio de Janeiro (UNIRIO), Rio de Janeiro, Brazil; Pos Graduate Program in Neurology (PPGNEURO), Federal University of the State of Rio de Janeiro (UNIRIO), Rio de Janeiro, Brazil

**Keywords:** Crohn’s disease, extra-intestinal manifestations of inflammatory bowel diseases, sustained attention, neuropsychological assessment, cognitive impairment

## Abstract

**Background:**

Behavioral and cognitive manifestations have been increasingly recognized in Crohn disease (CD), yet the specific behavioral domains associated with clinical activity remain incompletely characterized. Although reduced response speed has been proposed as a key feature, moment-to-moment fluctuations in performance may better capture behavioral instability related to systemic inflammation. We examined behavioral variability and attentional performance in CD and their relationship with clinical activity.

**Methods:**

In this case–control study, patients with CD of at least 1 year’s duration (*n* = 31) and healthy controls (*n* = 37) completed a short, computerized attention test assessing sustained-attention, focused-attention, inhibitory control, and processing speed. Group differences were evaluated using multivariate and univariate analyses of covariance. Binary logistic regression evaluated the discriminative ability of attentional measures, while correlations and regression analyses examined associations with disease activity measured by the Harvey-Bradshaw Index (HBI).

**Results:**

Fifty percent of patients with CD were in clinical remission. Compared with controls, patients with CD demonstrated significantly greater attentional variability and higher omission error rates, whereas mean response speed did not differ significantly. Within the CD cohort, sustained attention was significantly associated with disease activity and was the only subdomain predicting HBI scores. Patients in clinical remission continued to show impairments in sustained and focused attention compared with controls, whereas response speed was unaffected.

**Conclusion:**

CD is associated with selective alterations in attentional stability rather than generalized slowing. Attentional variability tracks clinical activity and remains detectable during remission, suggesting that sustained attention may represent a sensitive cognitive marker of ongoing disease impact.

## Introduction

Crohn’s disease (CD), one of the major forms of inflammatory bowel disease (IBD), is characterized by a chronic, noninfectious inflammatory process that can affect any segment of the gastrointestinal tract, from the oral cavity to the anus.^1^ Recent global estimates indicate that the burden of IBD remains high, with a worldwide prevalence of 229.7 cases per 100 000 inhabitants (95% CI: 212.4-247.0). Specifically, CD shows a prevalence of 84.2 cases per 100 000 inhabitants (95% CI: 78.5-89.9), with the highest rates observed in Europe and North America.^2^ However, its incidence has been rising in newly industrialized regions such as Africa, Asia, and Latin America,^3^ a trend likely associated with environmental changes. In Brazil, for example, CD has shown an annual increase of higher than 1.1% (95% CI: 4.8-17.8).^4^

Growing evidence indicates that CD is associated with alterations in the gut–brain axis,^5^ which may contribute to cognitive dysfunction in some patients.^6^ Previous studies have reported cognitive impairment in individuals with CD, including reduced processing speed,^7^ as well as deficits observed during both active disease and remission.^8–10^ However, these findings alone do not clearly establish which cognitive domain is primarily affected. Because processing speed reflects only one aspect of attentional functioning rather than a broader cognitive construct, it may not fully explain the cognitive difficulties observed in this population. To our knowledge, the literature has not yet clearly identified the core cognitive domain underlying these impairments, highlighting the need for more comprehensive assessments of attentional functioning.

Attention is a core component of cognition and is essential for efficient memory and executive functioning.^11^ The full version of the Continuous Visual Attention Test (CVAT) has been widely employed in clinical research to assess attention subdomains, including processing speed.^12^^,^^13^ This test measures 4 subdomains of attention: sustained attention, focused attention, inhibitory control, and processing speed (reaction time [RT]). However, in clinical settings and large-scale studies, the length of the full version (approximately 15 minutes) presents limitations, particularly for individuals with IBD, chronic pain, or fatigue, for whom prolonged cognitive effort can hinder performance.

To overcome these limitations, an ultra-brief 90-second version of the CVAT was developed. Comparative analyses between the ultra-brief and the original full-length versions demonstrated significant correlations for all corresponding attention parameters, providing evidence of concurrent validity between the two versions. Moreover, sustained attention showed not only a significant correlation but also agreement across versions.^14^ Additionally, the ultra-brief CVAT has demonstrated validity and sensitivity across different clinical contexts, maintaining its ability to discriminate attentional profiles.^15–17^

The present study used the ultra-brief CVAT to assess attentional performance in patients with CD, including those in clinical remission and moderate disease, compared with demographically matched healthy controls. Our first aim was to conduct a domain-specific analysis of attention. The second objective was to test whether these neuropsychological measures could discriminate between patients and controls. For the third objective, we conducted an exploratory analysis to identify which subdomains of attention most strongly predicted disease severity in the patient cohort. Finally (fourth objective), we examined differences in sustained attention, processing speed, and error patterns on the CVAT between healthy controls and patients with CD, stratified by disease activity (remission vs active disease), while controlling for age and sex.

## Methods

### Participants

Two groups of participants were recruited. (1) CD group: patients presenting with CD of at least 1 year’s duration. (2) Control group: pain-free volunteers with no history of IBD. Demographic data (age, sex) and current medication use were collected.

#### Inclusion criteria (Crohn’s disease cohort)

**Figure 1:**
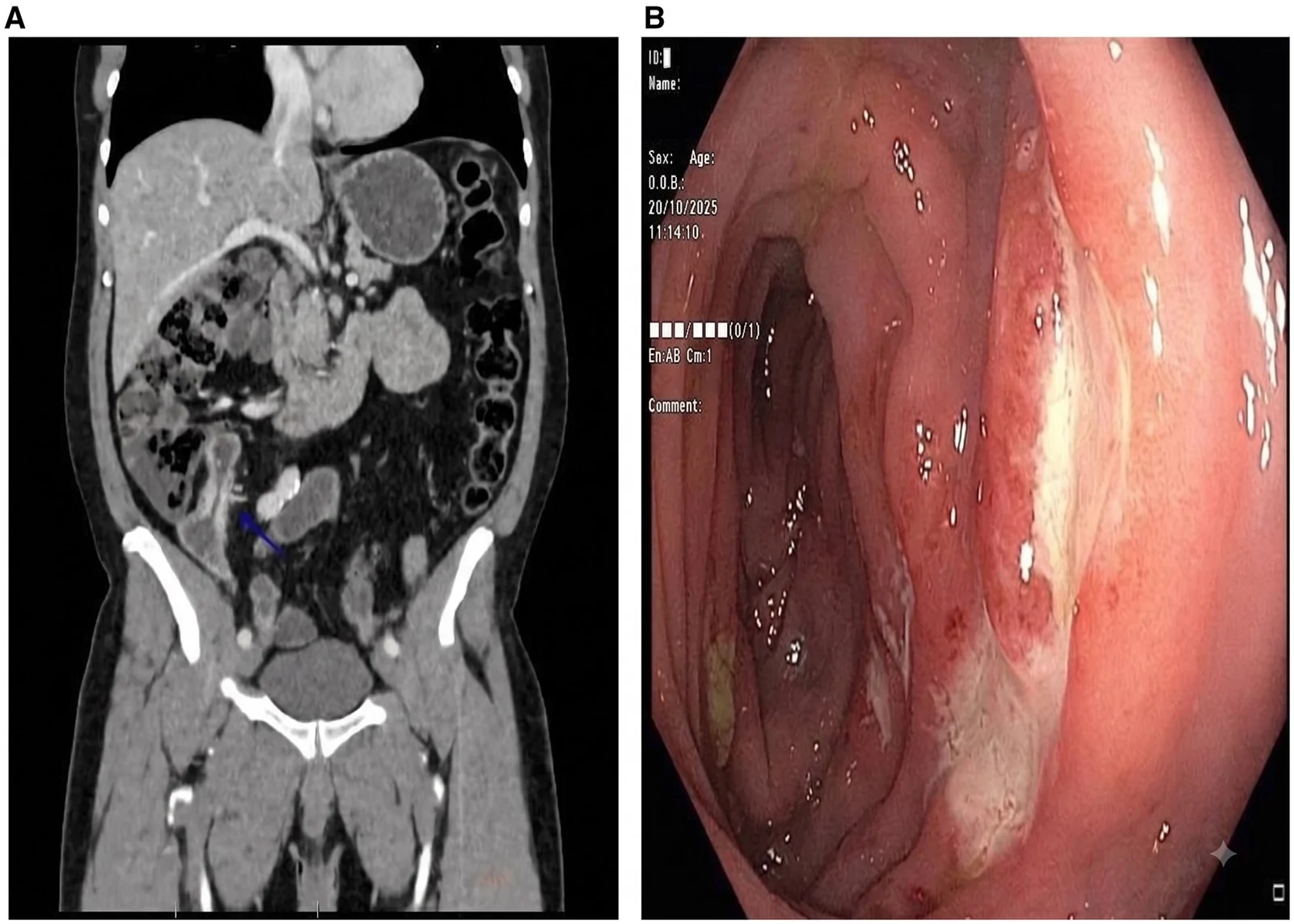
(A) Enterotomography of a patient with Crohn’s disease. Involvement of a jejunal loop, showing predominantly symmetrical, stratified bowel wall thickening, some segments associated with luminal stenosis with upstream dilatation, interspersed with segments of normal wall thickness. A segment in the right iliac fossa, with an estimated length of 3.2 cm and wall thickness of up to 9 mm. (B) Colonoscopy demonstrates a serpiginous ulcer with an edematous and hyperemic border, deep, with a fibrin-covered base measuring approximately 13 mm at its greatest axis, located in the transverse colon. In this case, the patient presented ulcers with similar characteristics and varying sizes in all segments of the large intestine and in the terminal ileum.

The diagnosis of CD was established based on a multifaceted approach, reflecting the condition’s complex pathogenesis. Clinical assessments included the evaluation of gastrointestinal symptoms like abdominal pain and diarrhea with or without blood or mucus, alongside extra-intestinal manifestations such as uveitis and arthralgia. Biochemical testing, including full blood counts, inflammatory markers, and metabolic profiles (iron, B12, liver, lipids, and thyroid function), was conducted to assess disease activity, nutritional status, and common findings like anemia. Additionally, stool studies (fecal calprotectin and *Clostridioides difficile* toxin) were utilized to quantify inflammation and exclude infectious mimics. The definitive diagnosis was confirmed through the integration of cross-sectional imaging, specifically computerized tomography enterography for transmural evaluation (Figure 1A) or ileocolonoscopy (Figure 1B) with biopsy. Endoscopic and histopathological findings included skip lesions, cobblestone patterns, strictures, crypt architectural distortion and non-caseating granulomas, a crucial finding for the differential diagnosis with intestinal tuberculosis, particularly within the Brazilian epidemiological context.

#### Exclusion criteria (controls and patients)

1. Neurologic disease affecting cognition/attention (eg, dementia, stroke, moderate–severe Traumatic Brain Injury, Multiple Sclerosis, Parkinson’s, epilepsy).
2. Active major psychiatric disorders associated with attentional impairment (eg, major depression, bipolar, schizophrenia).
3. Attention-altering medications within 30 days (eg, stimulants, benzodiazepines, antipsychotics, chronic opioids, high-dose antidepressants, pregabalin/gabapentin).
4. Substance use disorder (alcohol or illicit) currently active.
5. Rheumatologic disease.
6. Recent traumatic brain injury or surgery
7. Cancer.
8. Task limitations: Severe motor impairment, uncorrected visual deficits, or inability to complete a valid CVAT practice trial

### Procedures

This was a prospective, case–control study designed to evaluate the performance of CD patients compared with healthy controls in the 90-second CVAT.

#### Harvey-Bradshaw Index

For all participants with CD, clinical disease activity was quantified using the Harvey-Bradshaw Index (HBI), a validated, simplified proxy for the CD Activity Index. The HBI score, derived from patient-reported general well-being, abdominal pain, daily number of liquid stools, the presence of an abdominal mass (assessed by a clinician), and extra-intestinal complications, was calculated for each patient. Based on established cutoffs, patients were classified into the following clinical states: clinical remission (HBI ≤ 4), mild activity (HBI 5-7), moderate activity (HBI 8-12), risk of progressing to severe disease (13-16), and severe activity (HBI > 16).

#### Continuous Visual Attention Test

**Figure 2:**
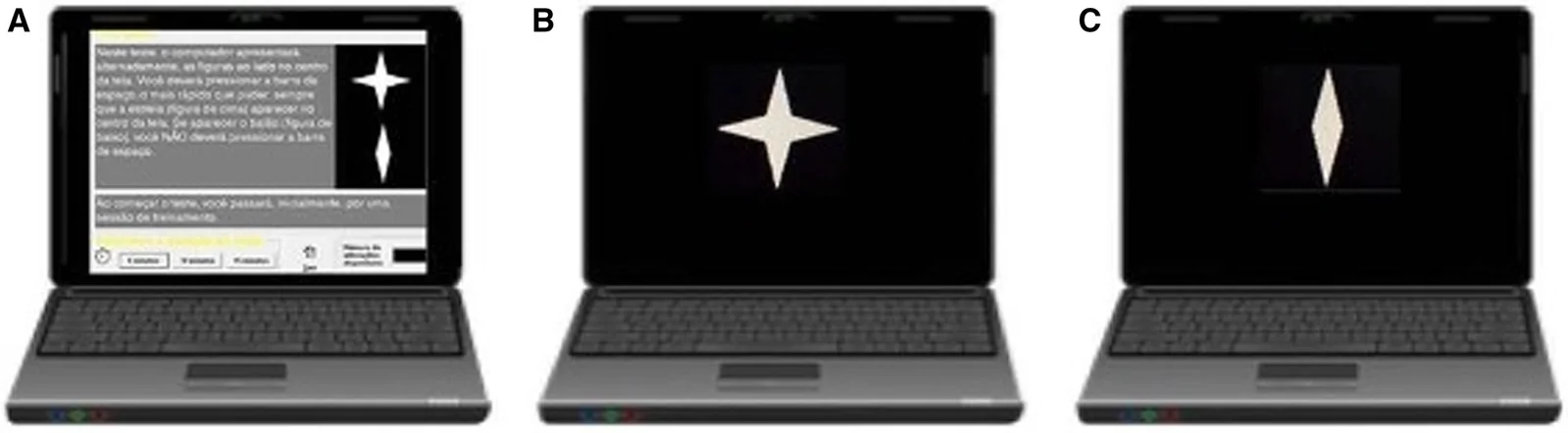
Computerized Visual Attention Test (CVAT). Instruction screen presented prior to task initiation (A). Participants were instructed to press the spacebar whenever the target stimulus (B) appeared in the center of the screen and to withhold response when the non-target stimulus (C) was presented.

The CVAT is a continuous computerized test based on a Go/No-Go paradigm (Figure 2). Participants were positioned approximately 50 cm from the monitor and instructed to press the spacebar with their dominant hand as quickly as possible in response to the target stimulus, while inhibiting their response to the non-target stimulus. In the event of an omission or commission error (CE), participants were instructed not to interrupt the task. Before the test began, a training phase was administered. Participants proceeded to the actual task only after completing the training without errors, demonstrating that they had understood the instructions. The task consisted of 72 target stimuli (80%) and 18 non-target stimuli (20%), presented in pseudorandom order. Each stimulus was displayed for 250 milliseconds, followed by a fixed interstimulus interval of 750 milliseconds. The duration of the task was 90 seconds.

The CVAT provides 4 main performance variables: (1) RT: mean latency of correct responses (alertness/processing speed); (2) Variability of Reaction Time (VRT): intraindividual standard deviation of RTs (sustained attention); (3) omission errors (OEs): failures to respond to target stimuli (focused attention); (4) CEs: incorrect responses to non-target stimuli (inhibitory control). A secondary measure, the coefficient of variability (CV = VRT/RT), was also calculated and analyzed.

### Statistical analysis

Prior to modeling, statistical assumptions were evaluated: normality (skewness/kurtosis and *Q*–*Q* plots), homogeneity of covariance matrices (Box’s test), and homogeneity of error variances (Levene’s test). All analyses were conducted with a two-tailed *α* = 0.05.

#### Sample size calculation

Sample size was determined for a two-sided comparison of means between two independent groups, assuming a type I error rate (*α*) of 0.05 and 80% power. Clinically meaningful differences were defined a priori based on normative data, participant age and sex distributions, and published reference values. The minimum detectable differences (D) were set to correspond to effect sizes considered clinically relevant: OE, *D* = 3 errors; CE, *D* = 5 errors; RT, *D* = 60 ms; and VRT, *D* = 25 ms. Under these assumptions, analyses for each parameter of the CVAT indicated that a minimum of 11 participants per group was required to detect the specified mean differences, assuming equal group sizes.

Because unequal group enrolment was anticipated, statistical power was preserved by increasing the total sample size according to the expected allocation ratio. Assuming a 2:1 allocation ratio, a total sample size of 42 participants (14 in the smaller group and 28 in the larger group) was required to maintain 80% power.

Sample size calculations were based on unadjusted comparisons of group means. Age and sex were included as covariates in the primary analyses to improve precision and account for potential confounding. No additional inflation of the sample size was applied, as covariate adjustment is expected to reduce residual variance.

For the regression analyses, the minimum number of participants per variable is 10.

##### First objective

MANCOVA was performed to compare the two groups, with age and sex included as covariates. The comparison between patients with CD and healthy controls focused on CVAT performance measures: OE, CE, mean RT, and VRT. When assumptions were violated (eg, homogeneity of covariance matrices), Pillai’s trace was used as the primary multivariate statistic due to its robustness.

When multivariate MANCOVA was significant, ANCOVAs with Bonferroni correction were performed for each CVAT variable: RT, VRT, OE, and CE. For effect size interpretations, partial *η*² from the ANCOVAs, and Cohen’s d for between-group differences were reported.

Additionally, to examine group differences in attentional performance, nonparametric analyses were conducted using the Mann–Whitney U test, which served as a confirmatory analysis for the group mean differences due to the non-normal distribution of the dependent variables.

To determine whether the effects of variability were independent of response speed, the MANCOVA, respective ANCOVAs, and Mann–Whitney tests were repeated using the coefficient of variability (VRT/RT) instead of VRT and RT.

##### Second objective

A binary logistic regression was performed to evaluate whether sex, age, and CVAT performance (OE, CE, RT, and VRT) predicted the presence of CD (0 = no disease; 1 = CD). Additional models were run replacing RT and VRT with the coefficient of variability (VRT/RT), which represents VRT independently of mean response time.

##### Third objective

To examine the contribution of CVAT performance variables to CD activity, we conducted 2 complementary analytic approaches. Our goal was to determine how disease severity, as assessed by the HBI, relates to performance and to identify the strongest cognitive predictors of disease activity.

Given our small sample size, all analyses were considered exploratory. First, we computed both parametric and non-parametric correlations between HBI scores and all CVAT indices (CE, OEs, RT, and VRT).

We then performed multiple linear regression analyses using a forward selection method to identify the best predictors of disease activity. We initially entered all 4 original CVAT variables into the model as well as age and sex.

Finally, to specifically evaluate the role of attention variability independent of reaction speed, we constructed a second model using a generalized linear approach. In this model, RT and VRT were replaced by the coefficient of variability (CV = VRT/RT). This substitution allowed us to test whether intra-individual variability, rather than mean speed, is more strongly associated with CD activity.

##### Fourth objective

We followed the same procedures described in the first objective. Group differences between controls, CD in remission, and CD with active disease were examined using a General Linear Model (MANCOVA) with disease group as the fixed factor and age and sex as covariates. When multivariate MANCOVA was significant, ANCOVAs with Bonferroni correction were performed for each CVAT variable: RT, VRT, OE, and CE. To determine whether the effects of variability were independent of response speed, the MANCOVA and respective ANCOVAs were repeated using the coefficient of variability (VRT/RT) instead of VRT and RT. Multivariate effects were assessed using Pillai’s trace. Bonferroni-adjusted pairwise comparisons were based on estimated marginal means. Statistical significance was set at *P* < .05.

## Results

### Demographic and clinical characteristics

A total of 68 participants were analyzed (Table 1), including 37 individuals in the Control Group (without CD) and 31 in the Patient Group (with CD). The demographic profile of 31 patients with a confirmed diagnosis of CD revealed a slight female predominance, with 16 (51.6%) female and 15 (48.4%) male patients. The mean age of the sample was 41.7 years. Disease duration, assessed based on patient-reported symptom onset, was available for 27 of the 31 patients. The data indicated a population with long-standing disease, with a median duration of 6 years.

**Table 1:** Participant demographics and CVAT performance in controls and Crohn’s disease patients in remission and active disease.

Classification	Subjects	Demography		Performance				
**Sample size**	68	Age, mean ± SD	Sex (% female)	OE, mean ± SD	CE, mean ± SD	RT (ms), mean ± SD	VRT (ms), mean ± SD	CV, mean ± SD
**Control group**	37	40.1 ± 15.23	51.4%	0.35 ± 0.16	3.32 ± 0.47	374.92 ± 6.14	67.38 ± 3.29	0.18 ± 0.01
**Crohn group**	31	41.7 ± 15.16	51.6%	2.94 ± 0.73	4.52 ± 0.50	408.48 ± 12.11	103.03 ± 7.95	0.25 ± 0.01
**Remission (HBI ≤ 4)**	16	37.4 ± 13.4	50.0%	2.00 ± 0.92	4.81 ± 0.63	382.31 ± 13.78	86.88 ± 8.21	0.22 ± 0.01
**Active disease (HBI ≥5)**	15	46.3 ± 16.0	53.3%	5.72 ± 1.09	4.20 ± 0.81	436.40 ± 18.03	120.27 ± 12.73	0.27 ± 0.02

Demographic: SD, HBI, and performance: OE, CE, VRT, RT and CV. CV = VRT/RT, SE.

Abbreviations: CE, commission errors; CV, coefficient of variability; HBI, Harvey-Bradshaw Index; OE, omission errors; RT, reaction time; SD, standard deviation; SE, standard error; VRT, variability of reaction time.

Disease activity was assessed in all CD patients (Table 1). The median HBI observed was 4.5. As shown in Table 1, 51.6% of patients (*n* = 16) were classified as being in clinical remission at the time of data collection. The maximum HBI score observed was 9, confirming that all patients presented with an HBI < 10, indicating that no patient met the criteria (>12) for severe disease activity or the need for hospitalization at the time of assessment. The treatment profile of the sample (*N* = 31) was analyzed (Table 2) focusing on the use of biologic therapy. The cohort was relatively balanced regarding treatment. Biological therapy was used either as monotherapy in 25.8% of patients (*n* = 8) or as combination therapy in 35.5% (*n* = 11). A total of 32.3% of patients (*n* = 10) were not using biological agents but were receiving other pharmacological treatments. Only 6.5% of the patients (*n* = 2) were not receiving any medication at the time of evaluation.

**Table 2:** Medications in use (Crohn’s disease patients).

Treatment category	Medications in use	Number	%
**Biological therapy (monotherapy)**	Infliximab; adalimumab	8	25.8
**Biological therapy (combination therapy)**	Infliximab + azathioprine; adalimumab + azathioprine	11	35.5
**No biological therapy (other medications only)**	Azathioprine; mesalazine; symptomatic or supportive medications	10	32.3
**No medication**	None	2	6.5
**Total**	—	31	100

### CVAT performance—descriptive statistics

Regarding task performance, the mean number of OE was 0.35 ± 0.16 (mean ± Standard error of the mean) in controls and 2.94 ± 0.73 in CD patients. Similarly, the mean number of CE was 3.32 ± 0.47 in controls and 4.52 ± 0.50 in patients, with both groups showing approximately normal distributions.

The mean RT for correct responses was substantially faster in controls, 374.92 ± 6.14 ms than in CD patients 408.48 ± 12.11 ms, indicating slowed processing in individuals with CD. Variability of RT (VRT) was also higher in CD patients 103.03 ± 7.95 ms than in controls 67.38 ± 3.29 ms, reflecting reduced attentional consistency in the Crohn’s group. A similar pattern was observed for the coefficient of variability (VRT/RT), which was 0.18 ± 0.01 in controls and 0.25 ± 0.01 in CD patients.

Overall, patients with CD presented a higher number of errors, slower responses, and greater intraindividual variability than participants without CD.

#### First objective: group differences in measures of central tendency

**Figure 3:**
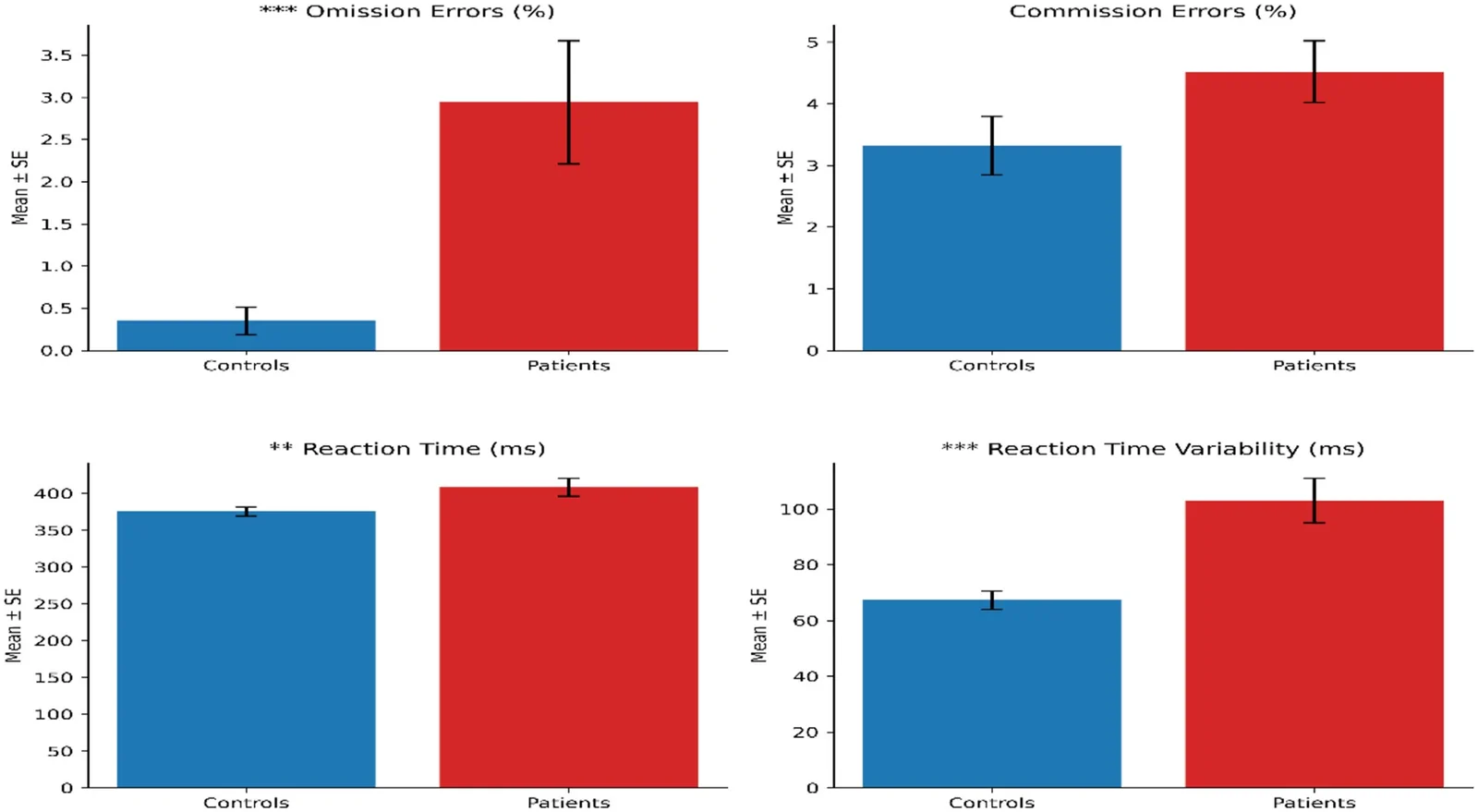
Patients show higher omission errors, slower responses, and higher variability than controls. **P < .01, ***P < .001, *P*, *P* value after correcting for multiple comparisons; SE, standard error.

MANCOVA results indicated significant group differences in attentional performance (Pillai’s trace = 0.296, *F* (4,61) = 6.414, *P* < .001, partial *η*² = 0.296) (Figure 3). The covariates age and sex also showed significant multivariate effects. ANCOVAs revealed the following results: OE: *F* (1, 64) = 14.60, *P* < .001, *η*² = 0.19; CE: *F* (1, 64) = 2.77, *P* = .10, *η*² = 0.042; RT: *F* (1, 64) = 7.31, *P* = .009, *η*² = 0.103; VRT: *F* (1, 64) = 25.70, *P* < .001, *η*² = 0.287.

When VRT and RT were replaced by the coefficient of variability (ie, VRT divided by RT), the same pattern of results was observed. The multivariate analyses, based on Pillai’s trace, revealed a significant main effect of group (Pillai’s trace = 0.299, *F* (3, 62) = 8.80, *P* < .001, *η*² = 0.299). Similarly, age and sex influence the outcomes in this analysis. Subsequent univariate ANCOVAs confirmed significant group difference in OEs *F* (1, 64) = 14.60, *P* < .001, *η*² = 0.186) and the coefficient of variability *F* (1, 64) = 24.05, *P* < .001, *η*² = 0.273). CE did not reach significance.

Nonparametric analyses using the Mann–Whitney U test confirmed significant group differences for the variables: OE: *U* = 277.50, *Z* = −4.176, *P* < .001; VRT *U* = 257.50, *Z* = −3.89, *P* < .001, and VRT/RT: *U* = 263.50, *Z* = −3.817, *P* < .001.

Taken together, these results demonstrate that individuals with Crohn’s disease exhibited slower, more variable, and less accurate attentional performance compared with the controls. However, the persistence of group differences in the coefficient of variability indicated that the effect of Crohn disease on VRT (sustained attention) is at least partially independent of RT.

#### Second objective: binary logistic analyses

Results using binary logistic regressions indicated that when all predictors were entered simultaneously (sex, age, OE, CE, RT, VRT), the model was statistically significant, *χ*²(6) = 29.67, *P* < .001, indicating that the predictors reliably distinguished participants with and without CD. The model explained between 36% (Cox & Snell *R*²) and 47% (Nagelkerke *R*²) of the variance and correctly classified 75% of cases. The Hosmer–Lemeshow test was non-significant, *χ*²(8) = 11.70, *P* = .17, suggesting good model fit. The most reliable predictor was VRT (Wald = 5.96, *P* = .015, Exp(B) = 1.059) (Table 3).

**Table 3:** Binary logistic regression analysis identifying variables that discriminate healthy controls from patients.

Variables	*B*	SE	Wald	Sig.
**OE**	0.338	0.272	1.547	0.214
**CE**	0.036	0.145	0.062	0.803
**RT**	0.001	0.011	0.008	0.930
***VRT***	***0.057***	***0.024***	***5.956***	***0.015***
**Age**	0.036	0.025	2.152	0.142
**Sex**	1.279	0.750	2.913	0.088

All predictors (sex, age, OE, CE, RT, and VRT) were entered simultaneously into the model. Regression coefficients (*B*), SE, Wald statistics, and significance levels (Sig.) are reported. VRT emerged as the only significant predictor and is highlighted in bold. RT, OE, and CE did not reach significance.

Abbreviations: CE, commission errors; OE, omission errors; RT, reaction time; SD, standard deviation; SE, standard error; VRT, variability of reaction time.

Then, we performed a binary logistic regression replacing RT and VRT with CV (VRT/RT) to determine whether attentional performance (OE, CE, and CV) age, and sex predicted the likelihood of CD. Using the Enter method, the full model was statistically significant (*χ*²(5) = 27.379, *P* < .001). The model explained 33% of the variance (Cox & Snell R²) and 44% (Nagelkerke *R*²) and demonstrated good calibration (Hosmer–Lemeshow *χ*²(8) = 3.778, *P* = .877). The classification accuracy was 72.1%. Within this model, only coefficient of variability (VRT/RT) emerged as a significant predictor (*B* = 22.168, Wald = 6.812, *P* = .009), with higher coefficient of variability associated with substantially increased odds of CD. The remaining predictors—OE, CE, age, and sex—did not reach statistical significance.

#### Third objective: regressions between CVAT variables and disease progression

**Figure 4:**
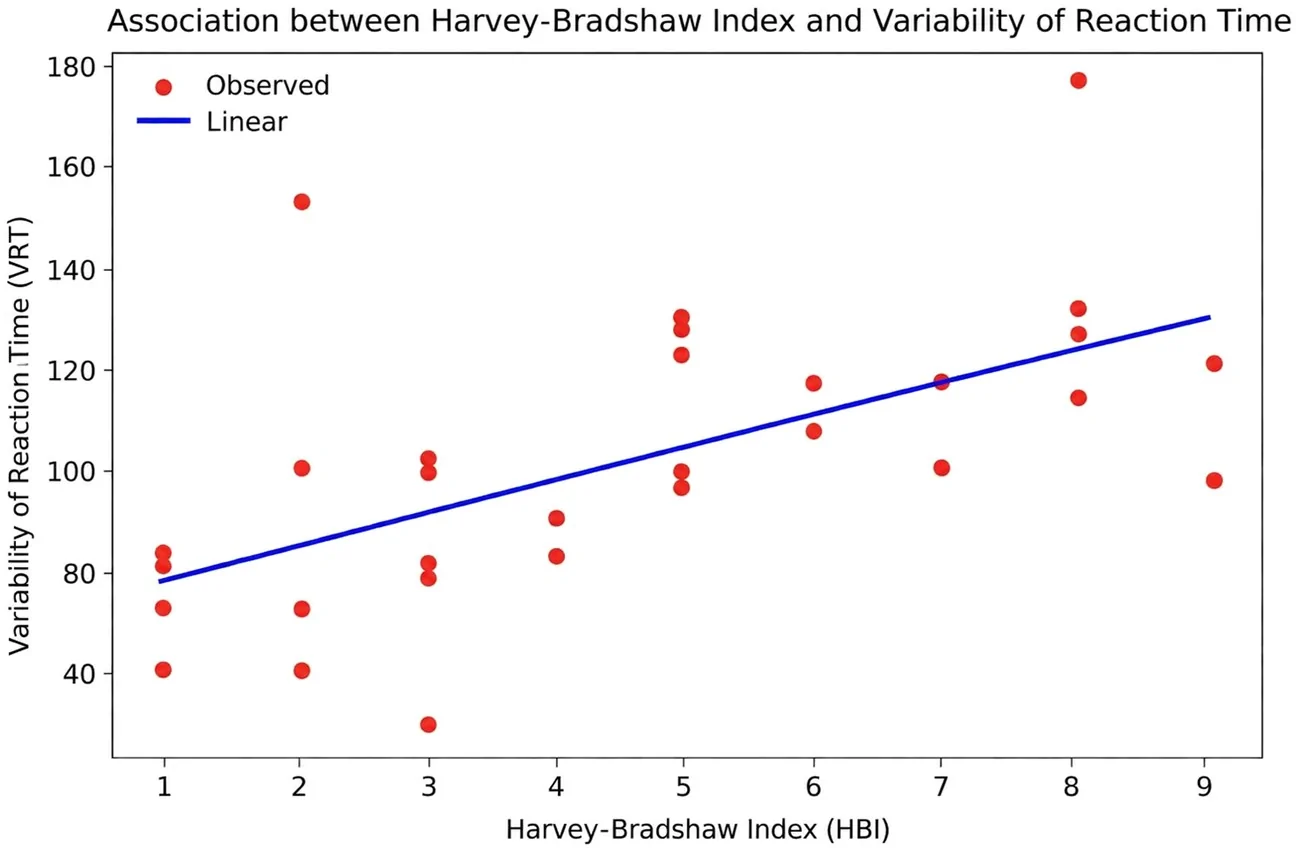
The analysis of the association between disease activity (HBI) and variability of reaction time (VRT) indicates that sustained attention decreases (ie, VRT increase) when clinical disease activity increases. Multiple linear regression analysis confirmed that VRT—not error rates or mean speed—is the single strongest predictor of clinical disease activity.

Pearson correlations showed that the HBI was significantly associated with RT (*r* = 0.45, *P* = .012) and variability of RT (VRT) (*r* = 0.48, *P* = .006) (Figure 4). In contrast, OE (*r* = 0.28, *P* = .131) and CE (*r* = −0.022, *P* = .906) did not correlate with HBI. The pattern was confirmed by Spearman tests, with significant associations for RT (*ρ*  =  0.36, *P* = .044) and VRT (*ρ*  =  0.40, *P* = .028), and no significant associations for OE or CE. These correlations suggest that slower RTs and increased temporal instability are the CVAT dimensions most closely related to disease activity.

When RT and VRT were replaced by the coefficient of variability (CV), HBI correlated significantly with CV (*r* = 0.42, *P* = .018), whereas OE and CE again showed no association with disease activity. Spearman tests confirmed this pattern (CV: *ρ* = 0.40, *P* = .024). These results indicate that CV captures the same clinical signal as VRT, despite correcting for the dependency of VRT on mean RT.

A forward linear regression was conducted with HBI as the dependent variable and age, sex, OE, CE, mean RT, and VRT as candidate predictors. The procedure identified VRT as the only variable meeting entry criteria. The final model was significant, *F* (1, 29) = 8.62, *P* = .006, explaining 23.0% of the variance in disease activity (*R*² = 0.23; adjusted *R*² = 0.20). Greater variability in RT predicted higher levels of CD activity (*β* = 0.48, *P* = .006). None of the other variables—age, sex, OE, CE, or RT—met criteria for entry (all *P* ≥ .29). This analysis shows that, among classical CVAT parameters, temporal instability (VRT) was the single strongest predictor of clinical disease activity.

A second forward regression replaced RT-based variables with the coefficient of variability (CV). Candidate predictors were age, sex, OE, CE, and CV. Again, only CV met criteria for entry. The final model was significant, *F* (1, 29) = 6.45, *P* = .018, explaining 18% of the variance in HBI (*R*² = 0.18; adjusted *R*² = 0.15). Higher CV values were associated with higher disease activity (*β* = 0.424, *P* = .018). Age, sex, RT, OE, and CE were not retained (all *P* ≥ .25).

Thus, even when controlling for the dependency of variability on mean RT, variability-based measures remained the only significant predictor of CD activity, strengthening the evidence that attention instability—not error rates or mean speed—is the cognitive dimension most tied to inflammatory status.

#### Fourth objective: CVAT performance according to Crohn’s disease activity

Descriptively, mean RT and VRT increased progressively from controls to patients in remission and were highest in patients with active CD, whereas OEs were more frequent in both CD groups than in controls (Table 3). In the General Linear Model controlling for age and sex, a significant overall group effect was observed on CVAT performance (Pillai’s trace = 0.405, *F*(8, 122) = 3.87, *P* < .001). Univariate analyses showed significant group effects for mean RT (*F*(2, 63) = 7.35, *P* = .001) and VRT (*F*(2, 63) = 17.82, *P* < .001), with Bonferroni-adjusted comparisons indicating slower RTs and greater variability in patients with active disease compared with controls, while patients in remission did not differ from controls in RT but showed higher variability (*P* = .024). OEs also differed significantly across groups (*F*(2, 63) = 8.63, *P* < .001), with patients in remission exhibiting more OEs than controls, whereas CE did not differ significantly between groups (*F*(2, 63) = 1.70, *P* = .190).

When VRT and RT were replaced by the coefficient of variability (ie, VRT divided by RT), the same pattern of results was observed. The multivariate analyses, based on Pillai’s trace, revealed a significant main effect of group and subsequent univariate ANCOVAs confirmed significant group differences in OEs and the coefficient of variability. CE did not reach significance.

### Ethical considerations

Written informed consent was obtained from each participant. This study was approved by the local Ethics Committee (CAAE: 7 1166823.2.0000.5258), which was conducted in accordance with the Declaration of Helsinki.

## Discussion

When analyzing measures of central tendency, the CD group exhibited slower, more variable, and less accurate attentional performance compared with the control group. Logistic regression indicated that the model adequately discriminated patients with CD from controls, with VRT emerging as the key predictor. In analyses restricted to the CD cohort, linear regression identified VRT as the primary predictor of disease activity. Furthermore, VRT and OE, but not RT, were impaired in patients in remission as compared to controls.

Our findings regarding an increase in RT in the patient group corroborate previous literature with respect to slower processing speed RT. Indeed, cognitive alterations in CD have been widely discussed across several studies^5^^,^^6^^,^^18^^,^^19^ with processing speed often identified as a sensitive marker of the disease.^8^^,^^9^ In addition, the observed impairments in VRT and OE represent novel contributions. Deficits in OE and VRT were also found in hospitalized patients with COVID-19 presenting gastrointestinal symptoms.^17^ As VRT and OE are related to sustained attention and attentional lapses,^17^ we suggest that attentional instability (VRT) and reduced focused attention may reflect shared pathophysiological mechanisms associated with gastrointestinal symptoms.

The analysis derived from the logistic regression demonstrates that sustained attention is the domain that best discriminates CD patients from controls. Importantly, VRT emerges as an independent predictor independent of processing speed, as demonstrated by the coefficient of variability. These results suggest that cognitive alterations in CD are not limited to a global slowing of processing speed, but rather involve dynamic failures in maintaining attentional states over time, consistent with momentary attentional lapses. Furthermore, the results based on the coefficient of variability indicate that attentional impairment is more strongly related to VRT than to processing speed.

The present results on the association between disease activity and the variables of the CVAT demonstrate that VRT shows the most robust association with disease activity and is at least partially independent of mean RT. Previous studies have reported cognitive impairment in CD and associations between disease severity, systemic inflammation, and objective cognitive slowing.^10^^,^^20^ Given that most prior studies have focused on average response times without examining variability, our findings suggest that attentional instability, rather than global slowing of processing speed, may represent the primary cognitive alteration in CD.

Another relevant finding was the demonstration of VRT and OE impairments in remission, indicating that these variables remain affected even in contexts of low inflammatory activity. This pattern may help explain cognitive alterations reported in other domains, such as memory and executive functions, in patients in clinical remission.^7^^,^^10^ Replacing VRT and RT with the coefficient of variability (VRT/RT) further reinforced that the discriminative power of VRT is, at least in part, independent of mean response speed. Deficits in sustained and focused attention may persist even in patients in clinical remission, possibly reflecting mechanisms related to low-grade chronic inflammation and dysregulation of the microbiota–gut–brain axis.^21^^,^^22^

Patients with CD frequently report experiencing brain fog, characterized by impaired concentration, reduced mental clarity, and difficulties sustaining attention.^23^ Although this symptom has only recently begun to be systematically investigated, its underlying cognitive mechanisms remain poorly understood. A recent study demonstrated that brain fog is highly prevalent among individuals with IBD, affecting more than 90% of participants,^23^ and is independently associated with greater psychological distress and poorer quality of life.^23^^,^^24^ Our findings provide objective evidence of significant attentional deficits in patients with CD, thereby supporting the validity of these commonly reported cognitive complaints. Together with previous studies demonstrating measurable cognitive impairment in CD, including deficits in processing speed and other cognitive domains,^7–10^ our results suggest that attentional dysfunction may represent an important neurocognitive substrate underlying patients’ subjective experience of brain fog. From a clinical perspective, these findings may help IBD clinicians recognize that patients’ cognitive complaints reflect measurable alterations in attentional functioning rather than solely subjective experiences or psychological distress. Objectively validating these symptoms may improve clinician–patient communication, facilitate a more comprehensive clinical assessment, and encourage discussions regarding strategies to minimize the impact of cognitive difficulties on daily functioning.

Although our study did not evaluate therapeutic interventions, our findings have important implications for future research. Cognitive rehabilitation programs targeting attentional functioning have been shown to improve objective measures of attention in neurological and neuropsychiatric disorders, including adults with attention-deficit/hyperactivity disorder^25^ and long-COVID brain fog.^26^ Moreover, a randomized controlled trial in patients with CD demonstrated that cognitive behavioral therapy and mindfulness-based interventions can modulate the brain–immune axis, supporting the potential responsiveness of cognitive symptoms to behavioral interventions.^27^ Although these findings cannot be directly generalized to attentional rehabilitation in CD, they suggest that attention-focused cognitive rehabilitation deserves further investigation as a potential strategy for alleviating brain fog and improving cognitive functioning. Future randomized controlled trials are warranted to determine whether such interventions can improve attentional performance, reduce the burden of brain fog, and ultimately enhance quality of life in patients with CD.

Several limitations of this study should be considered when interpreting the findings. First, the single-center design may restrict generalizability, particularly given the specialized nature of a tertiary academic hospital. Validation in multicenter cohorts encompassing different healthcare systems and patient populations will be essential to establish external validity. Second, although the overall sample size was modest (*n* = 68), with a limited number of patients in clinical remission (*n* = 16), this constraint is mitigated by using a neuropsychological task characterized by low intra-individual and normative variability. As detailed in the sample size calculations, this methodology allows for the reliable detection of clinically meaningful effects in relatively small samples. Finally, the cross-sectional design precludes conclusions regarding the directionality or temporal evolution of cognitive alterations in relation to disease activity, emphasizing the need for longitudinal studies. Additionally, as this was a cross-sectional study, causality cannot be inferred. Future studies should incorporate additional factors that may influence cognitive performance, including medical trauma, fatigue, sleep disturbances, and inflammatory biomarkers, all of which are commonly observed in patients with CD. Evaluating these variables may help clarify their contribution to attentional dysfunction and the experience of brain fog in this population.

Despite these limitations, the study has several strengths of particular relevance to IBD research and clinical practice. The cognitive assessment is brief (approximately 90 seconds), free of learning effects, and suitable for repeated administration, minimizing patient burden and facilitating implementation in both research and clinical settings.^15^ Importantly, the primary outcome measure, VRT, represents the most robust and reproducible parameter of the task. Consistent with prior work,^14^ VRT demonstrates strong concordance between full and abbreviated test versions and remains stable across variations in task parameters, including target frequency, stimulus presentation rate, and task duration. These characteristics support its reliability as an index of cognitive impairment in CD patients.

## Conclusion

In conclusion, from a clinical practice perspective, the identification of cognitive alterations in patients in clinical remission has important implications. Subclinical cognitive dysfunction may contribute to symptoms frequently reported by patients despite apparent disease control, such as cognitive fatigue, reduced concentration, and impaired daily functioning, which are not captured by conventional disease activity indices. Incorporating brief, objective cognitive measures into routine assessment may therefore provide complementary information on patient well-being and functional outcomes. Moreover, VRT emerges as a promising candidate biomarker for monitoring cognitive status and potentially treatment response over time. Longitudinal and interventional studies are now required to determine whether changes in VRT parallel disease course, inflammatory control, and therapeutic efficacy, and whether targeted interventions can ameliorate cognitive dysfunction in CD.

## Data Availability

Data are available upon request to the authors.
